# Dosimetric evaluation of cone beam computed tomography-guided online adaptive radiotherapy in gastric mucosa-associated lymphoid tissue lymphoma

**DOI:** 10.1016/j.tipsro.2025.100321

**Published:** 2025-06-25

**Authors:** Masanori Takaki, Taka-aki Hirose, Tadamasa Yoshitake, Keiji Matsumoto, Yuko Shirakawa, Hiroaki Wakiyama, Osamu Hisano, Hikaru Imafuku, Kousei Ishigami

**Affiliations:** a Department of Clinical Radiology, Graduate School of Medical Sciences, Kyushu University, 3-1-1 Maidashi, Higashi-Ku, Fukuoka 812-8582, Japan; b Division of Radiology, Department of Medical Technology, Kyushu University Hospital, 3-1-1 Maidashi, Higashi-Ku, Fukuoka 812-8582, Japan

**Keywords:** Online adaptive radiotherapy, Gastric MALT, CBCT, Intrafractional motion

## Abstract

**Introduction:**

This study evaluated dosimetric values of cone beam computed tomography (CBCT)-guided online adaptive radiotherapy (oART) in patients with gastric mucosa-associated lymphoid tissue (MALT) lymphoma, accounting for interfractional and intrafractional motion.

**Methods:**

Four patients with stage I gastric MALT lymphoma received CBCT-guided oART. For each of the 60 treatment sessions, scheduled (SCH) and adapted (ADP) plans were generated. Dosimetric evaluation focused on clinical target volume (CTV) and organs at risk (OARs). Metrics included CTV D98 % and D95 %, mean dose to the liver and left and right kidneys, maximum dose to the spinal cord, and V5Gy for bilateral kidneys. Adaptive planning CBCT-based contours were propagated to synthetic CTs of SCH and ADP plans to assess interfractional motion. Post-treatment CBCT-based contours were propagated to synthetic CTs of the ADP plan to evaluate intrafractional motion.

**Results:**

ADP plans significantly improved CTV coverage: mean D98% increased from 94.7 % in the SCH plan to 98.6 %, and D95% from 97.3 % to 99.2 % (p < 0.001). Most OAR doses were reduced in the ADP plans, including bilateral kidney V5Gy (11.3 % vs. 8.3 %, p < 0.001) and spinal cord Dmax (9.8 Gy vs. 7.9 Gy, p < 0.001). Liver Dmean was slightly higher in the ADP plan (11.4 Gy vs. 11.1 Gy, p = 0.002). No significant differences were observed in CTV and OAR dosimetric parameters between adaptive planning and post-treatment CBCTs (e.g., CTV D98%: 98.6 % vs. 98.5 %, p = 0.629).

**Conclusion:**

CBCT-guided oART improved target coverage and maintained post-treatment dosimetric stability in gastric MALT lymphoma, supporting clinical feasibility.

## Highlights

• The dosimetric values of CBCT-guided oART for gastric MALT lymphoma were evaluated with CBCT for oART and after irradiation
• CBCT-guided oART improved the target coverage, and improved dose distribution was maintained even after irradiation
• CBCT-guided oART may enable the precise targeting of irradiation in gastric MALT lymphoma

## Introduction

External beam radiotherapy effectively treats *Helicobacter pylori*-negative or eradication-refractory cases of localized gastric mucosa-associated lymphoid tissue (MALT) lymphoma [1,2], achieving a complete remission rate exceeding 99 % and a 10-year overall survival rate of approximately 90 %, indicating favorable treatment outcomes [3,4].

The stomach is a highly mobile and deformable organ. It undergoes inter- and intrafractional changes [5]. Online adaptive radiotherapy (oART) may be an effective approach for mitigating motion errors in such cases [6]. One limitation of oART is the prolonged treatment time compared to conventional image-guided radiotherapy. However, the Ethos system (Varian Medical Systems Inc., Palo Alto, CA, USA), a cone beam computed tomography (CBCT)-guided oART platform, enables clinically feasible oART by significantly reducing treatment time through artificial intelligence–driven auto-segmentation and automated treatment planning using the Intelligent Optimization Engine, thereby improving workflow efficiency compared to manual planning [[7], [8], [9]]. Countermeasures, including fasting, scopolamine butyl bromide administration, and breath-holding techniques, are implemented to address intrafractional motion [[10], [11], [12]]. Breath-hold techniques are effective in mitigating respiratory motion; however, they can prolong treatment time [13], potentially increasing intrafractional motion due to peristalsis. Unlike magnetic resonance-guided oART, which allows real-time monitoring during irradiation, CBCT-guided oART lacks this capability [14]. Therefore, in oART for MALT lymphoma, evaluating dose distribution while accounting for intrafractional motion is crucial.

Although only one study has validated the dosimetric value of CBCT-guided oART for gastric MALT lymphoma, considering intrafractional motion [15], that study used CBCT for oART and imaging immediately before irradiation. They reported a median irradiation time of approximately 11 min. According to Liu et al., even after excluding respiratory and cyclic gastrointestinal motions, the stomach exhibited slow drifting motion at a velocity of 0.7–1.4 mm/min [16]. Thus, irradiation times exceeding 10 min should not be considered negligible when evaluating intrafractional motion, and the significance of demonstrating that the dose distribution remains intact after irradiation is substantial. To our knowledge, no reports have evaluated planning and post-treatment CBCT. Therefore, this study aimed to evaluate the dosimetric values of oART for gastric MALT lymphoma using CBCT for oART and immediately after irradiation, considering interfractional and intrafractional motion.

## Material and methods

**Fig. 1:**
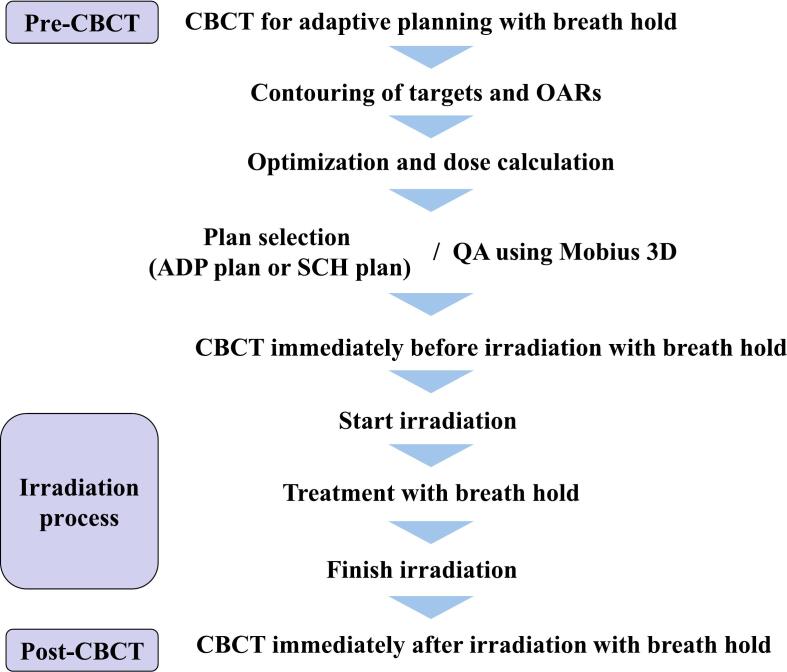
Workflow of online adaptive radiotherapy. QA, quality assurance; CBCT, cone beam computed tomography; OARs, organs at risk; ADP plan, adapted plan; SCH plan, scheduled plan.

This study was conducted in accordance with the principles of the Declaration of Helsinki. Ethical approval was obtained from the institutional review board of our hospital (Approval Number: 22077-00) on July 20, 2022. Written informed consent was obtained from all study participants to collect their clinical and planning data—including age, sex, clinical stage, treatment efficacy, adverse events, treatment planning details, and treatment duration—from electronic medical records or the treatment planning system to evaluate the efficacy of CBCT-guided oART. The study included four patients, two men and two women, of 50–70 years of age, who were diagnosed with gastric MALT lymphoma. All patients were classified as having stage-I disease according to the Lugano classification system. Prior to treatment, all patients fasted for at least 5 h and were administered scopolamine butylbromide 30 min before irradiation. The patients were instructed to hold their breath at the end of exhalation during image acquisition and irradiation. Breath-hold reproducibility was maintained within a gated window of ±2 mm using Breath Track (Engineering System Co., Ltd., Matsumoto, Nagano, Japan), which employs a charge-coupled device camera to detect an abdominal marker. Because Breath Track is an external device, CT imaging and irradiation were performed using a manually controlled gated window. The oART workflow is shown in Fig. 1.

### Reference (REF) plan

Planning computed tomography (CT) was performed using the Aquilion PRIME (Canon Medical Systems, Otawara, Japan) during a voluntary breath-hold at the end of normal expiration, with a slice thickness of 2 mm. CT scan was peformed twice. Contouring was conducted using Eclipse (version 16.1; Varian Medical Systems Inc., Palo Alto, CA, USA). The clinical target volume (CTV) encompassed the stomach and duodenal bulb, while the spinal canal, liver, and bilateral kidneys were designated as organs at risk (OARs) in the first set of CT images. To account for intrafractional motion, such as stomach peristalsis, the planning target volume (PTV) was defined by expanding the CTV by 10–15 mm ventrally, 20 mm to the left, and 10 mm in other directions, ensuring that the PTV encompassed both stomachs and duodenal bulbs identified on the second set of CT images. The data on the planning CT and contoured structures were imported into the Ethos planning system. A REF plan was created using 6 MV flattening filter-free photon beams and fixed-field intensity-modulated radiation therapy with 9 or 12 beams. A prescription dose of 30 Gy in 15 fractions over 3 weeks, which constituted the planned treatment period, was applied and normalized to ensure a PTV mean dose of 100 %. The optimization constraints are listed in Table 1. The optimization was performed to satisfy the clinical goals in order of their priorities, starting with those of highest priority. The following dosimetric parameters of the REF plans were calculated: CTV_ref_ D98% and D95%, liver_ref_ Dmean, spinal cord_ref_ Dmax, left kidney_ref_ Dmean, right kidney_ref_ Dmean, and bilateral kidneys_ref_ V5Gy. DX%, Dmean, Dmax, and VX% represent the dose covering X% of the target, the mean and maximum doses to OARs, and the volume of OARs receiving ≥ X Gy, respectively.

**Table 1:** Clinical goal for each structure.

Structure	Constraint				Priority
PTV	D95%	≥	100 %		1
PTV	Dmax	≤	105 %		1
Spinal cord + 0.3 cm	Dmax	≤	9 Gy		2
Left kidney	Dmean	≤	2 Gy	(5 Gy*)	2
Left kidney	D1%	≤	8 Gy		2
Right kidney	Dmean	≤	2 Gy	(5 Gy*)	2
Right kidney	D1%	≤	8 Gy		2
Liver − (PTV + 1 cm)	Dmean	≤	10 Gy	(15 Gy*)	2
Liver − (PTV + 1 cm)	D1%	≤	20 Gy		2

PTV, planning target volume; *Dose constraints varied for a single patient.

### Scheduled (SCH) and adapted (ADP) plans

On each treatment day, the atient was positioned supine and planning CBCT (pre-CBCT) was performed using the Ethos system. Artificial intelligence automatically delineated the stomach, liver, bilateral kidneys, and spinal canal on the CBCT image. The stomach contour was manually extended to include the duodenal bulb and the contours of the OARs were adjusted as needed.

The PTV was automatically generated by adding predefined margins to the stomach and duodenal bulb. The SCH plan was then recalculated using the synthetic CT, which was created by deforming the planning CT to match the pre-CBCT. An ADP plan was subsequently generated by reoptimizing the plan under the same conditions as the REF plan and recalculating it using the synthetic CT.

The dose distributions of the SCH and ADP plans were compared, and the plan with the superior distribution was selected for delivery. Immediately before irradiation, another CBCT image was recorded to ensure no significant deviations in the stomach outside the PTV or any other issues. Post-treatment CBCT (post-CBCT) was performed after irradiation to confirm that the CTV remained within the PTV.

### Retrospective evaluation of dose volume histogram parameters of SCH and ADP plans

For comparison, the data on 60 pre-and post-CBCT, SCH, and ADP plans were imported into Eclipse. To minimize intrafractional motion errors caused by prolonged treatment time, manual adjustments to the OAR contours on the day of the treatment were minimized. To correct the contouring errors the CTV (CTV_pre_) and OARs (liver_pre_, spinal cord_pre_, left kidney_pre_, and right kidney_pre_) for each treatment day were manually recontoured on the pre-CBCT in Eclipse. These contours were rigidly propagated to the synthetic CT from the pre-CBCT. For the SCH and ADP plans, the following dosimetric parameters were calculated: CTV_pre_ D98% and D95%, liver_pre_ Dmean, spinal cord_pre_ Dmax, left kidney_pre_ Dmean, right kidney_pre_ Dmean, and bilateral kidneys_pre_ V5Gy. In the oART workflow, as CTV_post_ and OARs_post_ are not automatically segmented on post-CBCT images, manual contouring was performed on the post-CBCT. The contours were then propagated to the synthetic CT of the ADP, and dosimetric values for CTV_post_, liver_post_, spinal cord_post_, left kidney_post_, and right kidney_post_ were calculated.

The CTV_pre_ and OARs_pre_ dosimetric parameters in the SCH plan reflect variations in REF plan dosimetry induced by interfractional motion. In contrast, the dosimetric parameters in the ADP plan represent values adjusted for interfractional motion through oART. Therefore, to evaluate the impact of oART on interfractional motion, differences in dosimetric parameters of CTV_pre_ and OARs_pre_ between the SCH and ADP plans were assessed. Differences between the dose parameters of CTV_pre_ and OAR_pre_ and those of CTV_pos_t and OAR_post_ in the ADP plan were considered to reflect the impact of intrafractional motion. Therefore, to determine whether intrafractional motion during the oART process remained within an acceptable range, the dosimetric parameters of CTV_pre_ and OARs_pre_ in the ADP plan were compared with those of CTV_post_ and OARs_post_.

### Treatment time

The time from the pre-CBCT acquisition to the completion of irradiation and the time from the start to the end of irradiation were recorded for each session.

### Statistical analysis

Statistical analyses were performed using Python (version 3.13.2; Python Software Foundation, Reston, VA, USA). The Shapiro-Wilk test and a linear mixed model were used to compare the dosimetric parameters between the plans. A P-value of <0.05 was considered statistically significant.

### Adverse events

Adverse events observed from the start of radiotherapy until the outpatient visit three months after completion of treatment were collected as acute adverse events. Acute adverse events were evaluated using the Common Terminology Criteria for Adverse Events, version 5.0.

## Results

CBCT-guided oART was completed within the planned period in all four cases. The ADP plan was adopted in all 60 treatment sessions. As acute adverse events, all cases exhibited grades 1–2 nausea; however, no grade ≥3 acute adverse events were observed. Complete remission was confirmed in all cases based on the primary efficacy assessment by upper gastrointestinal endoscopy performed three months after the completion of radiotherapy.

### Treatment time

The median time from pre-CBCT to the completion of irradiation was 29 min 8 s (interquartile range [IQR]: 25 min 46 s–32 min 41 s), and the irradiation time was 12 min 38 s (IQR: 11 min 10 s–15 min 3 s).

### Dosimetric values of SCH and ADP plans

Table 2 shows the mean dosimetric values and 95 % confidence interval of CTV_pre_ and OARs_pre_ in the SCH and ADP plans. The dosimetric values of the REF plan are also presented. The D98% and D95% for CTV_pre_ were significantly better in the ADP plan than in the SCH plan (p < 0.001). The Dmean of liver_pre_ was lower in the SCH plan (p = 0.002). There was no significant difference in the Dmean of the right kidney_pre_ between the SCH and ADP plans (p = 0.409). Other dosimetric values for OARs_pre_ were lower in the ADP plans (p < 0.001).

**Table 2:** Evaluation of interfractional motion using pre-CBCT dosimetric parameters between SCH and ADP plans.

	Planning CT in REF plan (n = 4)	Pre-CBCT in SCH plan (n = 60)		Pre-CBCT in ADP plan (n = 60)		
	Mean	Mean	(LCI–UCI)	Mean	(LCI–UCI)	P-Value
CTV D98% (%)	98.3	94.7	(92.4–97.0)	98.6	(98.4–98.8)	< 0.001
CTV D95% (%)	99.1	97.3	(96.2–98.3)	99.2	(99.1–99.2)	< 0.001
Liver Dmean (Gy)	11.1	11.1	(10.9–11.3)	11.4	(11.2–11.6)	0.002
Left kidney Dmean (Gy)	2.3	2.8	(2.3–3.3)	2.3	(1.9–2.7)	< 0.001
Right kidney Dmean (Gy)	1.9	2.1	(1.7–2.5)	2.1	(1.8–2.5)	0.409
Bilateral kidneys V5Gy (%)	8.7	11.3	(7.7–14.9)	8.3	(5.1–11.6)	< 0.001
Spinal cord Dmax (Gy)	7.9	9.8	(9.3–10.3)	7.9	(7.6–8.1)	< 0.001

CBCT, cone beam computed tomography; REF, reference; SCH, scheduled; ADP, adapted; LCI, lower 95% confidence interval; UCI, upper 95% confidence interval; CTV, clinical target volume.

Table 3 presents the comparison of the dose metrics for CTV_pre_ versus CTV_post_, and OAR_pre_ versus OAR_post,_ within the ADP plans. No significant differences were observed between the pre-CBCT and post-CBCT structures in any of the dose metrics, including CTV D98% (p = 0.629), CTV D95% (p = 0.484), liver Dmean (p = 0.233), left kidney Dmean (p = 0.319), right kidney Dmean (p = 0.770), bilateral kidneys V5Gy (p = 0.952), and spinal cord Dmax (p = 0.805).

**Table 3:** Evaluation of intrafractional motion based on differences in dosimetric parameters between pre- and post-CBCT in ADP plans.

	Pre-CBCT in ADP plan (n = 60)		Post-CBCT in ADP plan (n = 60)		
	Mean	(LCI–UCI)	Mean	(LCI–UCI)	P-Value
CTV D98% (%)	98.6	(98.4–98.8)	98.5	(98.3–98.7)	0.629
CTV D95% (%)	99.2	(99.1–99.2)	99.2	(99.1–99.3)	0.484
Liver Dmean (Gy)	11.4	(11.2–11.6)	11.3	(11.1–11.5)	0.233
Left kidney Dmean (Gy)	2.3	(1.9–2.7)	2.3	(1.9–2.7)	0.319
Right kidney Dmean (Gy)	2.1	(1.8–2.5)	2.1	(1.7–2.5)	0.770
Bilateral kidneys V5Gy (%)	8.3	(5.1–11.6)	8.3	(5.0–11.6)	0.952
Spinal cord Dmax (Gy)	7.9	(7.6–8.1)	7.8	(7.6–8.1)	0.805

CBCT, cone beam computed tomography; ADP, adapted; LCI, lower 95% confidence interval; UCI, upper 95% confidence interval; CTV, clinical target volume.

## Discussion

The purpose of this study was to evaluate changes in dosimetric parameters resulting from oART for gastric MALT lymphoma, considering interfractional and intrafractional motion. The CTV and OARs contoured on the pre-CBCT were propagated to the synthetic CTs of the SCH and ADP plans to calculate the dosimetric parameters. Target coverage was significantly improved with the ADP plan, indicating that interfractional motion was effectively mitigated. Most OARs also demonstrated more favorable dose metrics with the ADP plan, except for the liver_pre_ and right kidney_pre_. These exceptions may be attributed to the relatively lenient dose constraints applied to these organs and their lower optimization priority. Additionally, structures contoured on the post-CBCT were propagated onto the synthetic CT of the ADP plan to calculate the dosimetric parameters, which were then compared with those based on the pre-CBCT contours. No significant differences were observed in any of the dose metrics, suggesting that the degree of intrafractional motion between the pre-CBCT and the post-CBCT acquisitions remained within an acceptable range. Collectively, these findings indicate that CBCT-guided oART for gastric MALT lymphoma improved the target coverage.

To the best of our knowledge, this is the first study to demonstrate that improved dose distribution is maintained even after irradiation, as evidenced by post-treatment CBCT. Uto et al. reported that the ADP plan enhanced the dose coverage of the stomach during planning and pre-irradiation CBCT in patients with gastric MALT lymphoma [15]. They reported a median time of 30 min and 45 s (IQR: 25 min 2 s–32 min 39 s) from planning CBCT image acquisition to irradiation completion, with a median irradiation time of 11 min and 5 s. This suggests that the interval between planning CBCT and pre-treatment CBCT was approximately 20 min. In contrast, our study found that the median time from planning CBCT image acquisition to irradiation completion was 29 min 8 s (IQR: 25 min 46 s–32 min 41 s), with the interval between the planning CBCT and pre-treatment CBCT being approximately 17 min. Hirose et al. reported that the magnitude of intrafractional motion varied significantly between groups with intervals of <25 min and ≥35 min [17], indicating that CBCT acquired immediately after irradiation may be more suitable for evaluating intrafractional motion.

In this study, the PTV was created by adding margins of 10–15 mm ventrally, 20 mm to the left, and 10 mm in other directions to the CTV. This was adjusted considering the absence of solid organs, such as the kidneys and liver, on the left and ventral sides of the gastric body, which could lead to a greater intrafractional motion error due to the movement of gastrointestinal gas. The PTV was slightly larger than that reported in previous studies [15,18]. In studies on PTV margins for CBCT-guided oART in gastric MALT lymphoma, some have concluded that an 8 mm of uniform expansion around the CTV is appropriate [18], whereas others have suggested an expansion of 14 mm [17]. However, these studies were conducted with a small number of cases and are not universally applicable. Nevertheless, the potential of PTV reduction can be explored further.

Conventionally, irradiation with 30 Gy in 15–20 fractions is administered; however, recent reports have demonstrated good control with radiotherapy in a small number of fractions. Pinnx et al. reported no difference in outcomes between patients treated with standard radiotherapy doses of ≥30 Gy and those who received reduced radiotherapy doses of 24 Gy [19]. A pilot trial reported that response-adapted ultra-low dose radiotherapy might be useful as a definitive therapy. Most patients achieved a complete response following 4 Gy of radiotherapy, and those who required an additional 20 Gy achieved a complete response within 12 months [20]. For palliative treatment in older patients, 4 Gy in two fractions may also be viable [21]. As the number of irradiation fractions decreases, the impact of each fraction becomes more significant, increasing the importance of precise targeting. Therefore, oART may have substantial benefits.

Gastric MALT lymphoma generally has a favorable prognosis, making it important to pay attention to late toxicities. In previous reports on oART, the ADP plan had the lowest OARs dosimetric values [15,18]; however, in this study, only the liver_pre/post_ D_mean_ was the lowest in the SCH plan. This may be attributed to the lower priority assigned to liver dose constraints in our study. A study found that a V5Gy > 58 % for bilateral kidneys correlated with the incidence of late renal toxicity [22], suggesting that minimizing the dose to normal organs is crucial. Hirose et al. and Tison et al. reported that oART may reduce PTV margins [17,18]. By investigating the optimal PTV margin, achieving a balance between maintaining the dose to the target and minimizing the dose to OARs may be possible. Furthermore, with future improvements in CBCT quality, optimization, and calculation-time reduction, intrafractional motion is expected to decrease further.

The limitations of this study include its retrospective design, small sample size, and the inability to assess long-term outcomes. Additionally, the PTV margins varied slightly between cases. The values of CTV D_98%_ and D_95%_, which were used as indicators of plan accuracy, may change depending on the PTV margin, potentially introducing bias into the analysis.

## Conclusion

In patients with gastric MALT lymphoma, CBCT-guided oART significantly improved target coverage compared to the SCH plan, while maintaining a stable dose distribution even after irradiation. By effectively accounting for interfractional and intrafractional motion, CBCT-guided oART demonstrated high dosimetric precision and clinical feasibility.

## Declaration of Generative AI and AI-assisted technologies in the writing process

During the preparation of this work, the authors used　ChatGPT-4 (GPT-4-turbo) in order to translate Japanese into English. After using this tool/service, the authors reviewed and edited the content as needed and take full responsibility for the content of the publication.

## CRediT authorship contribution statement

**Masanori Takaki:** Conceptualization, Data curation, Investigation, Writing – original draft. **Taka-aki Hirose:** Data curation. **Tadamasa Yoshitake:** Writing – review & editing. **Keiji Matsumoto:** Supervision. **Yuko Shirakawa:** Supervision. **Hiroaki Wakiyama:** Supervision. **Osamu Hisano:** Supervision. **Hikaru Imafuku:** Supervision. **Kousei Ishigami:** Supervision.

## Funding

This study received funding by the Japan Society for the Promotion of Science (JSPS) KAKENHI (Grant Number JP19K17172 and JP25K10938).

## Declaration of competing interest

The authors declare that they have no known competing financial interests or personal relationships that could have appeared to influence the work reported in this paper.
